# ER stress activates TAp73α to promote colon cancer cell apoptosis via the PERK-ATF4 pathway

**DOI:** 10.7150/jca.84170

**Published:** 2023-07-03

**Authors:** Shengnan Sun, Yong Yi, Zhi-Xiong Jim Xiao, Hu Chen

**Affiliations:** 1 Center of Growth, Metabolism and Aging, College of Life Sciences, Sichuan University, Chengdu, 610064, China.; 2 Department of Cardiothoracic Surgery, The First Affiliated Hospital of Chengdu Medical College, Chengdu, 610500, China.; 3 Research Center of Translational Medicine, Central Hospital Affiliated to Shandong First Medical University, Jinan, Shandong, 250013, China.

**Keywords:** ER stress, Cell apoptosis, TAp73α, PERK, ATF4

## Abstract

Colorectal cancer (CRC) is the fourth most diagnosed cancer worldwide. 43% of CRCs harbor p53 mutations. The tumor suppressor p53 induces cell growth arrest and/or apoptosis in response to stress, including endoplasmic reticulum (ER) stress. It has been documented that the p53 gene is mutated in more than 50% of human tumors and loses its tumor suppressor function, suggesting that ER stress-induced apoptosis might not rely on p53. In this study, we found that activation of ER stress promotes p53 null colon cancer cell apoptosis concomitant with an increased level of the TAp73α protein, a homologue of p53 *in vitro* and *in vivo*. Knockdown of TAp73α partially restores ER stress-induced apoptosis, indicating that ER stress stimulates apoptosis in a manner dependent on TAp73α, but not p53. Furthermore, we found that ER stress activates TAp73α mRNA and protein expression through PERK signalling, a branch of the unfolded protein response (UPR). Moreover, PERK promotes TAp73α expression by upregulating the expression of the transcription factor ATF4. ATF4 directly activates the transcription of TAp73α. Consistent with this finding, ATF4 knockdown inhibited PERK- or ER stress-induced TAp73α expression. Our findings reveal that ER stress activates TAp73α to promote colon cancer cell apoptosis via the PERK-ATF4 signalling. Therefore, prolonged ER stress or upregulation of TAp73α might be a therapeutic strategy for colon cancer.

## Introduction

Colorectal cancer (CRC) comprises colon cancer and rectal cancer, in which colon cancer accounts for 71% of CRC cases and is the fourth most diagnosed cancer worldwide 1. Most CRCs are diagnosed in patients older than 50 years. Genetic and biobehavioral risk factors, including obesity, a sedentary lifestyle, high intake of red meat, alcohol consumption, and smoking, are major risk factors for CRC initiation. With the development of sequencing technology, increasing number of driver gene mutations have been found. The TP53 gene, which encodes the tumor suppressor protein p53, is mutated or deleted in more than 50% of human cancers and thus loses its tumor suppressor activity 2, 3. Of note, 43% of CRCs harbor p53 mutations (IARC TP53 database, R20; https://p53.iarc.fr/tp53SomaticMutations.aspx), of which approximately 90% mutations are missense mutations that impair wild-type p53 function (loss-of-function mutations) and might endow neo-morphic (gain-of-function mutations) to promote cancer progression 4.

The p53 protein is involved in all aspects of tumor initiation and development 5, 6. In addition, the p53 protein has two homologues: p63 and p73. In addition to performing functions partially overlapping with those of p53, p63 and p73 also play specific roles in tumor development. Since the DNA binding sites in p53 and p73 overlap, p73 also binds to and activates or represses p53 target genes that control apoptosis (e.g., PUMA, and BAX). Notably, the p73 protein is rarely mutated in human cancers and could compensate for the loss of p53. The TP73 gene, which encodes the p73 protein, harbours two different promoters that produce two major isoforms, TAp73 and ΔNp73. The TAp73 protein plays a role like that of wild type p53. In contrast, the N-terminal truncated ΔNp73 has an effect opposite to that of TAp73. It has been reported that TAp73α induces apoptosis by different mechanisms 7, one of which is ER stress. TAp73α can transactivate the expression of Scotin, a target protein of p53, to induce ER stress 8. However, the detailed role of TAp73α in ER stress-induced apoptosis is unknown.

During tumor initiation and progression, tumor cells are often exposed to different microenvironmental conditions, such as glucose deprivation, hypoxia, low pH, and DNA damage, which can result in the accumulation of unfolded or misfolded proteins in the endoplasmic reticulum (ER) lumen and consequently induce ER stress 9. Cells activate an adaptive mechanism in response to ER stress called the unfolded protein response (UPR). The UPR includes three parallel signalling branches: PRKR-like ER kinase (PERK)-ATF4, inositol-requiring protein 1α (IRE1α) - X-box binding protein (XBP1) and activating transcription factor 6α (ATF6α) 10. ER stress and UPR activation are documented to occur during the initiation and progression of many cancer types. Aside from its prosurvival role, prolonged ER stress or UPR activation leads to cell death owing to severe or unresolved ER stress 11, 12.

Herein, we demonstrated that ER stress activates TAp73α expression to promote colon cancer cell apoptosis that is not dependent on p53. Mechanistically, ER stress promotes TAp73α mRNA and protein expression through PERK-ATF4 signalling. Together, these results not only reveal the ER stress activates the PERK-ATF4-TAp73α signalling axis to promote apoptosis but also provide a new insight into the regulatory mechanism of TAp73α. Furthermore, these findings emphasize the potential of targeting TAp73α for cancer therapy.

## Materials and Methods

### Cell culture and generation of stable cell lines

HEK-293T cell and human colon cancer cell line HCT116 (p53 WT) were obtained from the American Type Culture Collection (ATCC). HCT116 (p53 Null) cell was generated by using CRISPR/Cas9, and donated as a kind gift from Dr. Dan Luo 13. HEK-293T cells were maintained in Dulbecco's modified Eagle's medium (DMEM), supplemented with 10% fetal bovine serum (PAN, Germany), 100 U/mL penicillin and 100 µg/mL streptomycin (GIBCO, Rockville, MD, USA). HCT116 cells were cultured in McCoy's 5A medium (Hyclone Inc, USA), supplemented with 10% fetal bovine serum (PAN, Germany), 100 U/mL penicillin and 100 µg/mL streptomycin (GIBCO, Rockville, MD, USA). All cells were maintained at 37 °C in a humidified incubator under 5% CO_2_.

Lentiviral particles were prepared as previously study 14. For the generation of stable cell lines, cells were infected with recombinant lentiviruses, then treated with 2 μg/mL puromycin (Sigma Inc, USA) at 48 h of post-infection for generation of stable cells expressing desired gene or shRNA.

### Plasmid construction

Human PERK, ATF4 and TAp73α genes were sub-cloned into pLVX-Puro vector. shRNA oligos were cloned into pLKO.1 vector according to protocol from Addgene instruction. shp73, shPERK, shIRE1α, shATF6α, and shATF4 oligos sequence are listed below. shp73-#1: 5'-CCCGCTCTTGAAGAAACTCTA-3', shp73-#2: 5'-ATCCGCGTGGAAGGCAATAAT-3'; shPERK-#1: 5'-GCAGGTCATTAGTAATTAT, shPERK-#2: CCGTAGTAAGAAATGGATCAT-3'; shIRE1α-#1: 5'-CTACTGGATAAACTTGCTTCA-3', shIRE1α-#2: 5'-GAGAAGATGATTGCGATGGAT-3'; shATF6α-#1: 5'-CTCGGTCAGTGGACTCTTA-3', shATF6α-#2: 5'-ACAGAGTCTCTCAGGTTAAAT-3'; shATF4-#1: 5'-ACCTTCTGACCACGTTGGAT-3', shATF4-#2: 5'-GTCCTCCACTCCAGATCATTC-3'; All plasmids used in this study were confirmed through DNA sequencing.

### Cell viability assay

The effect of ER stress inducers BFA (Brefeldin A; MCE; CAS No.: 20350-15-6) and TM (Tunicamycin; MCE; CAS No. :11089-65-9) or p73 on cell viability was measured by 3-(4,5-dimethylthiazol-2-yl)-2,5-diphenyltetrazolium bromide (MTS) assay. HCT116p^53+/+^ and HCT116^p53-/-^ cells were seeded into 96-well culture plate overnight. Cells were incubated with MTS (1 mg/ml) for 2 hours and then Optical density (OD) was measured using a microplate reader (Molecular Devices Co, San Jose) at 490 nm.

### Cell apoptosis assay

For apoptosis analysis, both suspended and attached HCT116 cells were collected gently in 100 μL and incubated with 5 μL FITC conjugated Annexin V and 5 μL PI (Vazyme Biotech) for 10 min at room temperature in dark. Cells were then subjected to FACS analysis by FACScan flow cytometer (Becton Dickson) and the data were analyzed using the Flow Jo software (Tree Star Inc., Ashland, Oregon). All the cells were gated and at least 20,000 cells were collected for each sample.

### Western blot analysis

Western blot analysis was used to detect protein expression and was performed as described previously 15. Antibody used in this study were as follows: p53 (1:2000, cat. sc-126, Santa Cruz, CA, US); p73 (1:1000, cat. 1636-1, Epitomics, US); GRP78 (1:1000, cat. 3183, Cell Signaling Technology (CST), Shanghai, China); PERK (1:1000, cat. 5683, CST); p-PERK (1:1000, cat. 340846, ZEN-BIOSCIENCE, China); ATF6α (1:1000, ab122897, Abcam, Shanghai, China), IRE1α (1:1000, cat. 3294, CST); ATF4 (1:1000, cat. 11815, CST); GAPDH (1:3000, cat. 301341, ZEN-BIOSCIENCE, China); Bim (1:1000, cat. 2933, CST); Caspase 3 (1:1000, cat. 9665, CST).

### Quantitative RT-PCR

Quantitative RT-PCR (qPCR) were used to detect the mRNA level and were performed as described previously 15. QPCR primer sequences are listed below: GAPDH-F: 5'-TGGACTCCACGACGTACTCA-3', GAPDH-R: 5'-AATCCCATCACCATCTTCCA-3'; GRP78-F: 5′-CATCACGCCGTCCTATGTCG-3′, GRP78-R: 5′-CGTCAAAGACCGTGTTCTCG-3; TP73-F: 5'-CACCACGTTTGAGCACCTCTGG-3', TP73-R: 5'-TGCTCAGCAGATTGAACTGGGC-3'; PERK-F: 5'-TGGCCACTTTGAACTTCGGTA-3', PERK-R: 5'-CCACCCGGTTTAAAGGTGCT-3'; ATF4-F: 5'-CAGCACAGCCCCTCTACCA-3', ATF4-R: 5'-GCCCGCCTTAGCCTTGTC-3'. Scotin-F: 5'-TGTGGAGCGAGGAAAGGTGTG-3', Scotin-R: 5'-CCCCTTATCCTCAGCCTCCAA-3'.

### Chromatin immunoprecipitation (ChIP) assay

ChIP assays were performed in HCT116^p53-/-^ cells, which were treated by TM (2μg/mL) for 24 hours, with ChIP-IT Kit (53009, Active Motif, USA) using antibodies specific for ATF4 (1:100, cat. 11815, CST) or normal rabbit IgG (1:100, cat. 2927, CST,), as described previously 14. ChIP samples were subjected to PCR experiments to amplify fragments of the TP73 and CHOP promoter elements using indicated primers as listed below: TP73-ChIP-F: 5'-CGTGTATGTAATGTATAAG-3', TP73-ChIP-R: 5'-CAGATGCGCGCGGGTGATG-3'; CHOP-ChIP-F: 5'-GATGGGACTGTCAGGAGTCTG-3', CHOP-ChIP-R: 5'-CTAGGGACTGGGCTTGGAAAG-3'; NC-ChIP-F: 5'-CATATCTGCTTGGGGTAC-3', NC-ChIP-R: 5'-GTGCAGTGTCTGAGCAAGTG-3'.

### Immunohistochemistry (IHC) assay

These paraffin-embedded slides were deparaffinized and rehydrated using xylene and a graded series of ethanol (100%, 95%, 80%, 75%), and then washed with PBS three times for 5 min each time. Subsequently, citrate antigen restore solution was used to repair antigens on slices in a microwave oven at the heat preservation for 15 min and followed by natural cooling, and washed with PBS three times for 5 min each time. It was then immersed in 3% H_2_O_2_ solution at room temperature to eliminate endogenous peroxidase activity, then incubated in 3% BSA to block non-specific binding of antibody for 1 h, incubated in a humidified chamber overnight at 4 °C with the primary antibodies, followed by washing with PBS and incubation with a secondary antibody for 60 min at room temperature. After washed with PBS, the slides were incubated with the DAB solution for staining. Subsequently, the slides were counterstained with hematoxylin, dehydrated with graded alcohol series, covered-slipped with neutral balsam and scanned through NanoZoomer (Hamamatsu, Japan).

### ER stress inhibits colon cancer growth *in vivo*

The patient-derived tumor xenograft (PDX) models were generated by direct transplantation of colon cancer patient tumor samples into immunocompromised NOD-scid IL2R gamma null (NSG) mouse, then the tumors were transplant to fifth to sixth-week-old BALB/c (nu/nu) mice. When the subcutaneous tumors attained a volume of 100 mm^3^ (day 0), the mice bearing tumor were divided into two groups, the mice in the treatment group were injected intraperitoneally (ip) tunicamycin 1 mg/kg (20 μg/mouse), three times a week, while the mice in the control group were received PBS only. The experiment was terminated and the tumor was removed after 3 weeks. Tumors were extracted for volume and weight measurement, and then fixed, embedded, and sectioned. Tumor sections were subjected to IHC staining for GRP78 (1:100, cat. 3177, CST), p73 (1:100, cat. Ab215038, Abcam), and Bim (1: 200, cat. 2933, CST). Written informed consent was obtained from the colon cancer patient and tumor specimens was approved by the Institutional Review Board at Chengdu Medical college. All animal experiments in this study were approved by the Animal Policy and Welfare Committee of Chengdu Medical College and the Institutional Animal Care and Use Committee (IACUC) of Sichuan University, and the procedures were performed according to the guidelines established by the China Council on Animal Care.

### Statistical analysis

All statistical analyses were performed using SPSS 16 software (SPSS Inc., Chicago, IL, USA). The data were presented as the mean ± SD. Comparisons between two groups were performed using the two-tailed unpaired Student's t-test. p < 0.05 were considered statistically significant.

## Results

### ER stress induces cancer cell apoptosis independent of p53

**Figure 1:**
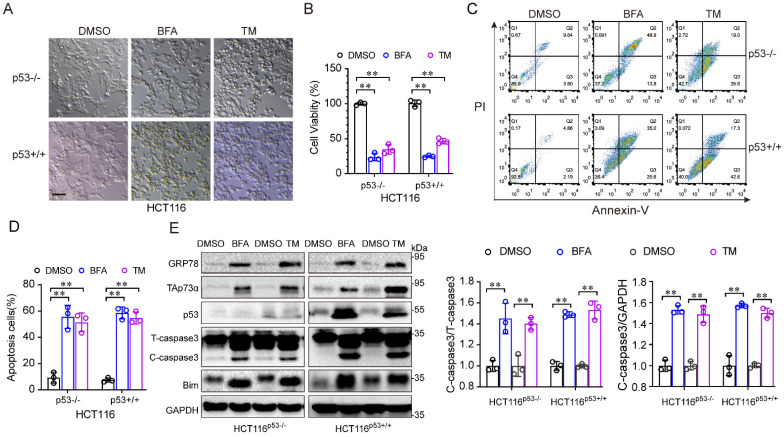
** ER stress induces cancer cell apoptosis independent of p53.** (A-E) The colorectal cancer cell line HCT116 with intact p53 (HCT116^p53+/+^) and loss of p53 (HCT116^p53-/-^) were treated with DMSO or 2 μg/mL BFA or TM (ER stress inducer) for 24 h. (A) Images of cell morphology were acquired by phase contrast microscopy. Scale bar = 100 μm. (B) Cell viability was measured by an MTS assay. (C-D) Cells were subjected to FACS for apoptosis detection (C) and quantification from three independent experiments (D). (E) Cells were subjected to western blot analysis of GRP78, TAp73α, p53 and apoptosis markers as indicated. and cleaved-caspase 3 (C-caspase3) were be quantified based on total-caspase 3 (T-caspase3) or GAPDH. The results are presented as the mean ± SD of three independent experiments; **P<0.01.

To investigate the role of p53 in ER stress, we treated HCT116 cells, in which the p53 protein was intact (HCT116^p53+/+^) or null (HCT116^p53-/-^) with ER stress inducers, such as, tunicamycin (TM) and brefeldin A (BFA). As shown in Fig. 1A, under ER stress inducer treatment, the morphology of both cell lines changed from an adherent, stretched cells to a wrinkled, round shape, with a tendency towards detachment and floating. In addition, the ER stress inducers significantly reduced the viability of both HCT116 cell lines (Fig. 1B). Moreover, apoptosis was obviously increased after ER stress inducer treatment (Fig. 1C-1D). Consistent with this result, the levels of apoptosis associated proteins, such as Bim and cleaved-caspase 3, were increased (Fig. 1E). Notably, the p53-family protein, TAp73α was upregulated in both cell lines after ER stress stimulation (Fig. 1E), indicating that TAp73α might involve in ER stress-induced cell apoptosis. Taken together, these findings demonstrate that ER stress promotes apoptosis independent of p53.

### ER stress promotes cancer cell apoptosis through TAp73α

**Figure 2:**
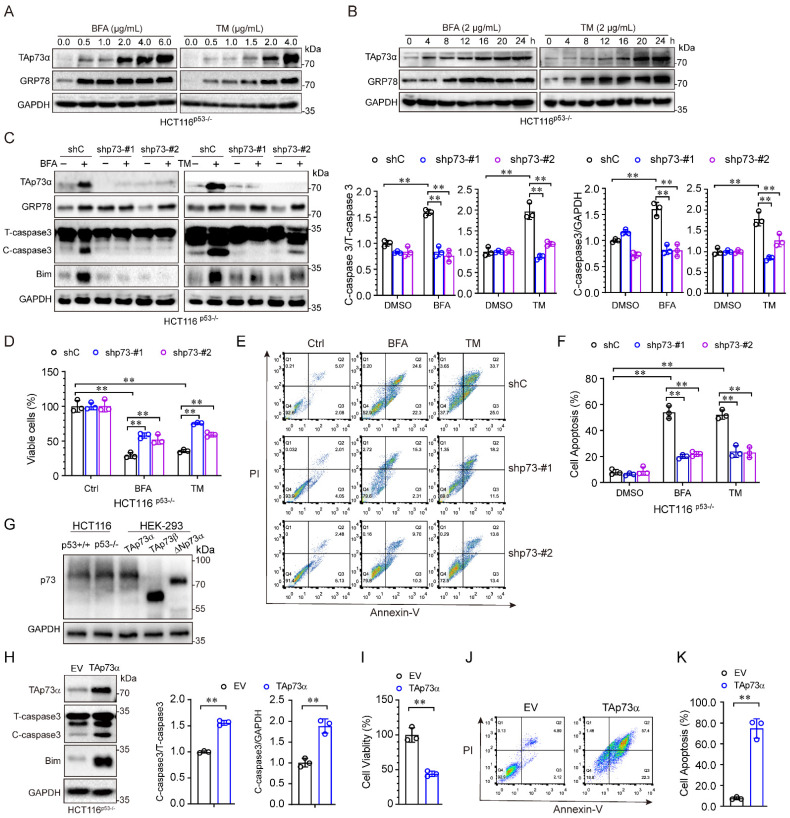
** ER stress induces cancer cell apoptosis via TAp73α.** (A-B) HCT116^p53-/-^ cells were treated with or without ER stress inducers (BFA and TM) at the indicated concentrations for 24 h (A) or a concentration of 2 μg/mL for different times (B) and then subjected to western blot analysis. (C-F) p53 null HCT116 cells stably expressing shC or shp73 were treated with or without ER stress inducers for 24 h, and then subjected to western blot analysis, in which cleaved-caspase 3 (C-caspase3) were be quantified based on total-caspase 3 (T-caspase3) or GAPDH (C), MTS assay (D) and FACS analysis (E), and quantitative analysis of FACS data from three independent experiments (F). (G) Whole cell lysates derived from HCT116^p53+/+^, HCT116^p53-/-^ cells and HEK-293T cells transiently transfected with TAp73α, TAp73β or ΔNp73α were subjected to western blot analysis. (H-K) HCT116^p53-/-^ stably expressing empty vector (EV) or TAp73α, and then subjected to western blot analysis, in which cleaved-caspase 3 (C-caspase3) were be quantified based on total-caspase 3 (T-caspase3) or GAPDH (H), MTS assay (I) and FACS analysis (J), and quantitative analysis of FACS data from three independent experiments (K). The results are presented as the mean ± SD of three independent experiments; **, P<0.01.

The aforementioned results showed that TAp73α expression was significantly upregulated by ER stress in p53 null cells. To investigate whether TAp73α is involved in ER stress induced apoptosis, we treated HCT116^p53-/-^ cells with different concentrations of ER stress inducers for different times. As shown in Fig. 2A, both of the ER stress inducers, BFA and TM, upregulated TAp73α expression in a dose-dependent manner. As expected, TAp73α protein expression also increased with prolonged ER stress treatment (Fig. 2B). To confirm whether TAp73α plays an important role in ER stress-induced cancer cell apoptosis, we knocked down TAp73α expression in p53 null HCT116 cells by using two specific p73 shRNAs. As shown in Fig. 2C, silencing TAp73α markedly impaired the upregulation of Bim and cleaved-caspase 3 induced by ER stress. Consistent with this finding, knockdown of TAp73α significantly reversed the ER stress-induced reduction in cell viability (Fig. 2D) and dramatically restored ER stress-induced apoptosis (Fig. 2E-2F). Given TAp73 proteins including several isoforms, we detected the isoforms of TAp73 in HCT116^p53+/+^ and HCT116^p53-/-^ cells, and found that TAp73α is the major isoform expressed in both HCT116 cells (Fig.2G). To further confirm whether TAp73α plays an important role in cell apoptosis, we ectopic expressed TAp73α in HCT116^p53-/-^ cells and found Bim and cleaved-caspase 3 upregulated obviously (Fig.2H). Consistently, ectopic expression of TAp73α significantly reduced cell viability (Fig. 2I) and dramatically promoted cell apoptosis (Fig. 2J-2K). Taken together, these findings demonstrate that TAp73α may play an important role in ER stress-induced cancer cell apoptosis.

### ER stress activates TAp73α through the PERK pathway

**Figure 3:**
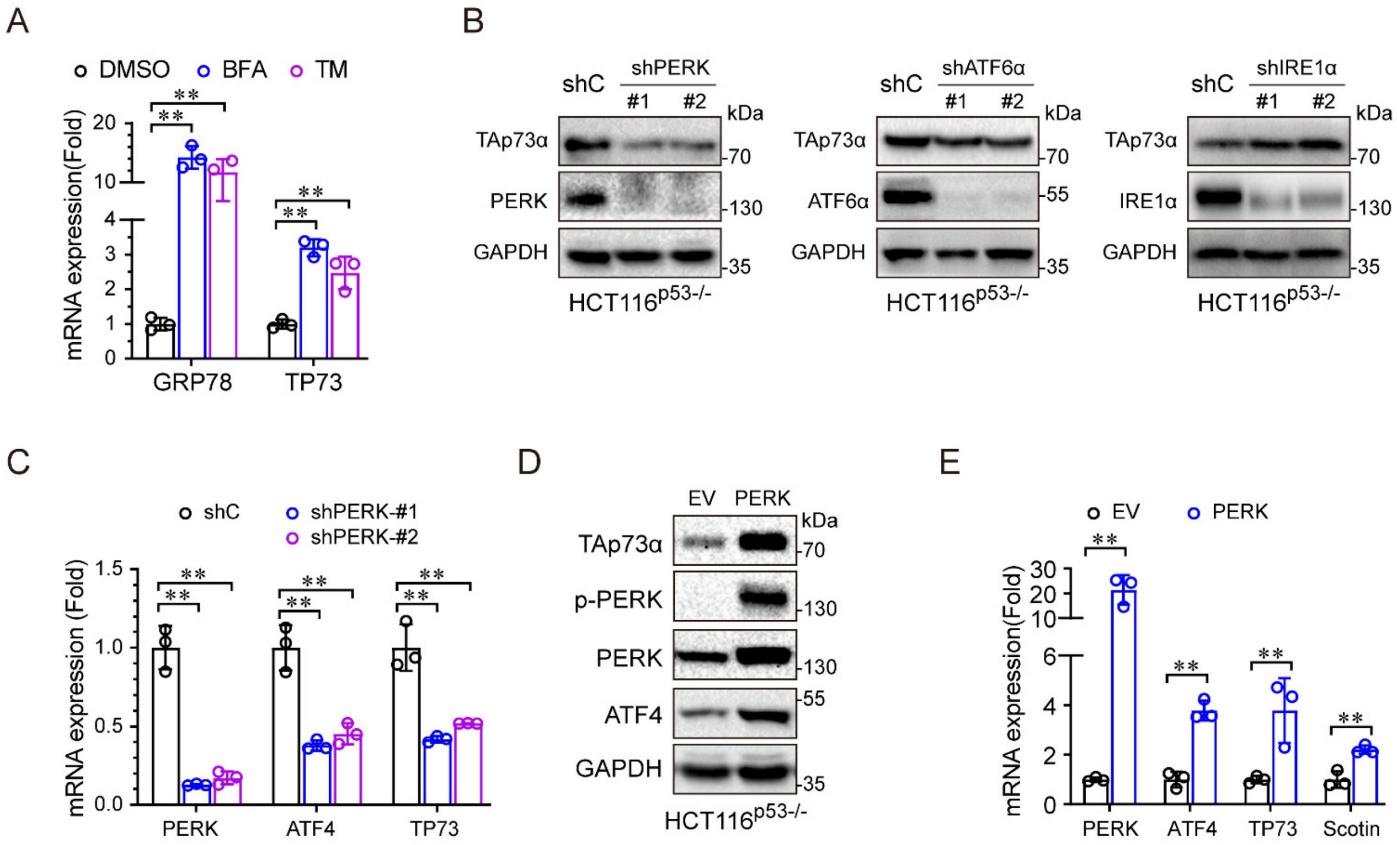
** ER stress activates TAp73α through the PERK pathway.** (A) HCT116^p53-/-^ cells were treated with or without ER stress inducers for 24 h and then subjected to qPCR analysis. (B) HCT116^p53-/-^ cells stably expressing shC, shPERK, shATF6α or shIRE1α were subjected to western blotting. (C) HCT116^p53-/-^ cells stably expressing shC or shPERK were subjected to qPCR analysis. (D-E) HCT116^p53-/-^ cells stably expressing empty vector (EV) or PERK were subjected to western blot analysis (D) and qPCR analysis (E). The results are presented as the mean ± SD of three independent experiments; **P<0.01.

We then investigated the molecular mechanism by which ER stress regulates TAp73α expression. During periods of ER stress, the three sensors, PERK, IRE1α and ATF6, dissociate from the ER membrane and activate gene expression to resolve ER stress. As shown in Figure 3A, the ER stress inducers (BFA and TM) significantly upregulated TP73 mRNA expression, indicating that ER stress might regulate TAp73α transcription. To investigate which ER stress signalling pathway is involved in regulating TAp73α expression, we separately knocked down PERK, ATF6 and IRE1α in HCT116^p53-/-^ cells and subjected them to ER stress stimulation. As shown in Figure 3B-3C, only knockdown of PERK significantly inhibited TAp73α protein expression, suggesting that ER stress might regulate TAp73α expression through PERK. Consistent with this finding, ectopic expression of PERK increased the protein levels of TAp73α and ATF4, the downstream effector or PERK (Fig. 3D). Moreover, PERK also upregulated the transcription levels of ATF4, TP73 and Scotin, the target of TAp73α (Fig. 3E). These data demonstrate that ER stress upregulates TAp73α expression through PERK signalling.

### ER stress upregulates TAp73α expression via the PERK-ATF4 axis

**Figure 4:**
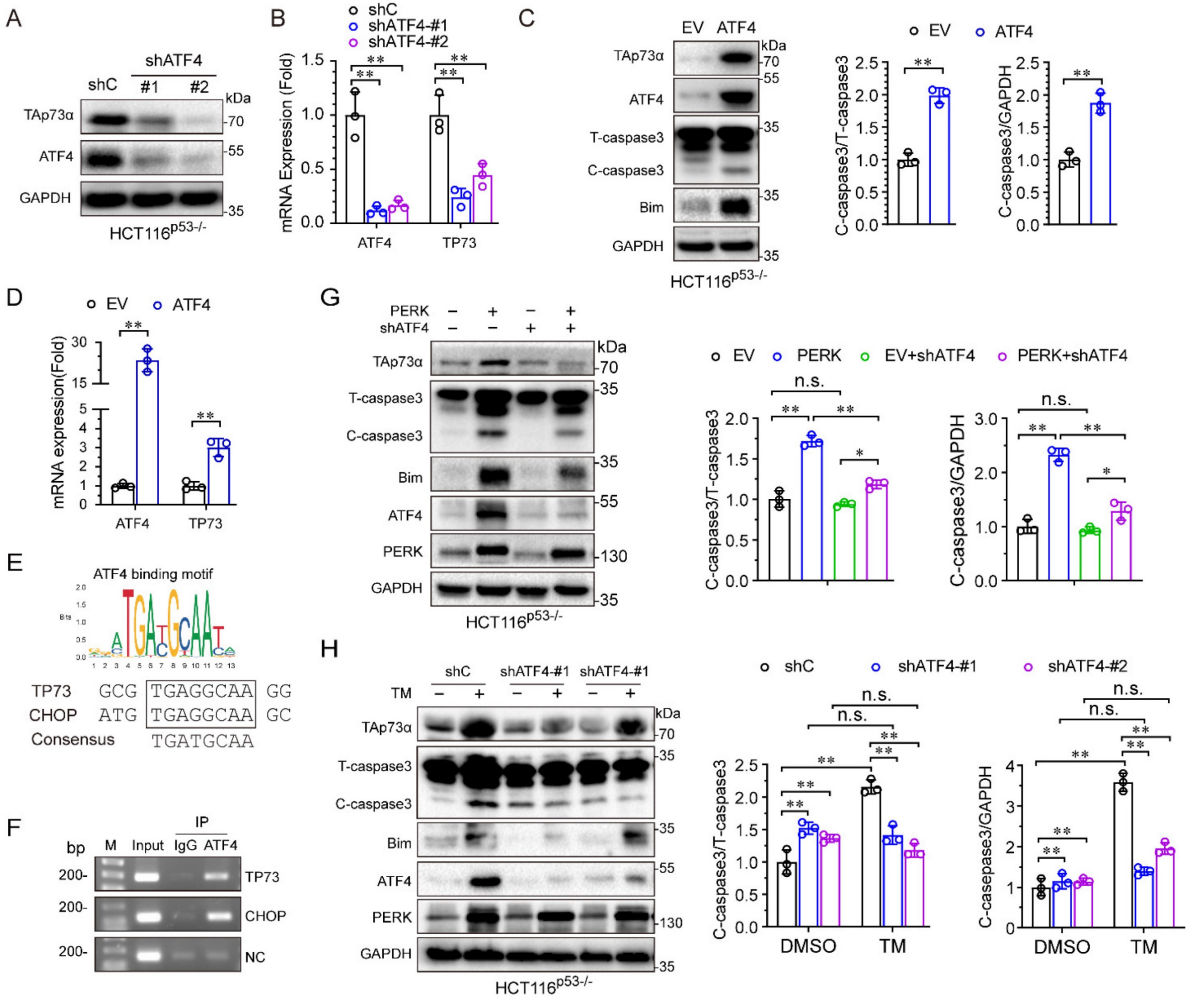
** PERK upregulates TAp73α expression through ATF4.** (A-B) HCT116^p53-/-^ cells stably expressing shC or shATF4 were subjected to western blot analysis and qPCR analysis (B). (C-D) HCT116^p53-/-^ cells stably expressing empty vector (EV) or ATF4 were subjected to western blot analysis, in which cleaved-caspase 3 (C-caspase3) were be quantified based on total-caspase 3 (T-caspase3) or GAPDH (C) and qPCR analysis (D). ATF4 binding motifs was presented (top panel). The putative binding sites of ATF4 on TP73 promoter were predicted by JASPAR (bottom panel) and compared with the binding site of ATF4 on CHOP promoter (E). HCT116^p53-/-^ cells were treated by TM (2 μg/mL) for 24 h, then subjected to ChIP analysis. (G) HCT116^p53-/-^ cells stably expressing PERK were transduced with lentiviral vectors encoding shATF4 or shC, and then subjected to western blot analysis, in which cleaved-caspase 3 (C-caspase3) were be quantified based on total-caspase 3 (T-caspase3) or GAPDH. (H) HCT116^p53-/-^ cells stably expressing shATF4 or shC were treated with or without TM for 24 h and then subjected to western blot analysis, in which cleaved-caspase 3 (C-caspase3) were be quantified based on total-caspase 3 (T-caspase3) or GAPDH. The results are presented as the mean ± SD of three independent experiments; **P<0.01.

During ER stress, PERK usually activates ATF4, and we speculated that ATF4 might be involve in the regulation of TAp73α by PERK. To address this hypothesis, we first silenced ATF4 in HCT116^p53-/-^ cells and found that both the protein and mRNA levels of TAp73α significantly decreased (Fig. 4A-4B). Second, we found that ectopic expression of ATF4 upregulated TAp73α expression (Fig. 4C-4D) and increased the levels of the pro-apoptotic proteins Bim and cleaved-caspase 3 (Fig. 4C), indicating that ATF4 could regulate TAp73α expression directly. Furthermore, *in silico* analysis of the TP73 promoter sequence by JASPAR (https://jaspar.genereg.net/) revealed a putative ATF4 binding sites (Fig. 4E). And ChIP assays showed ATF4 could bind to TP73 promoter and to the documented ATF4 binding site on the CHOP promoter 16. Together, these data demonstrate that ATF4 could direct activate the transcription of TAp73α.

To investigate whether PERK activates TAp73α expression through ATF4, we knocked down ATF4 under ectopic expression of PERK. As shown in Fig. 4G, knockdown of ATF4 significantly reduced the PERK-induced TAp73α upregulation as well as cleaved-caspase 3 and Bim expression. Furthermore, to confirm whether the ER stress-induced upregulation of TAp73α is dependent on ATF4, we knocked down ATF4 under ER stress stimulation condition. As shown in Fig. 4H, knockdown of ATF4 significantly reduced the ER stress-induced TAp73α upregulation as well as cleaved-caspase 3 and Bim expression. Taken together, these findings demonstrate that ER stress upregulates TAp73α expression via PERK-ATF4 signalling.

### Activation of ER stress inhibits colon cancer growth and promotes apoptosis *in vivo*

**Figure 5:**
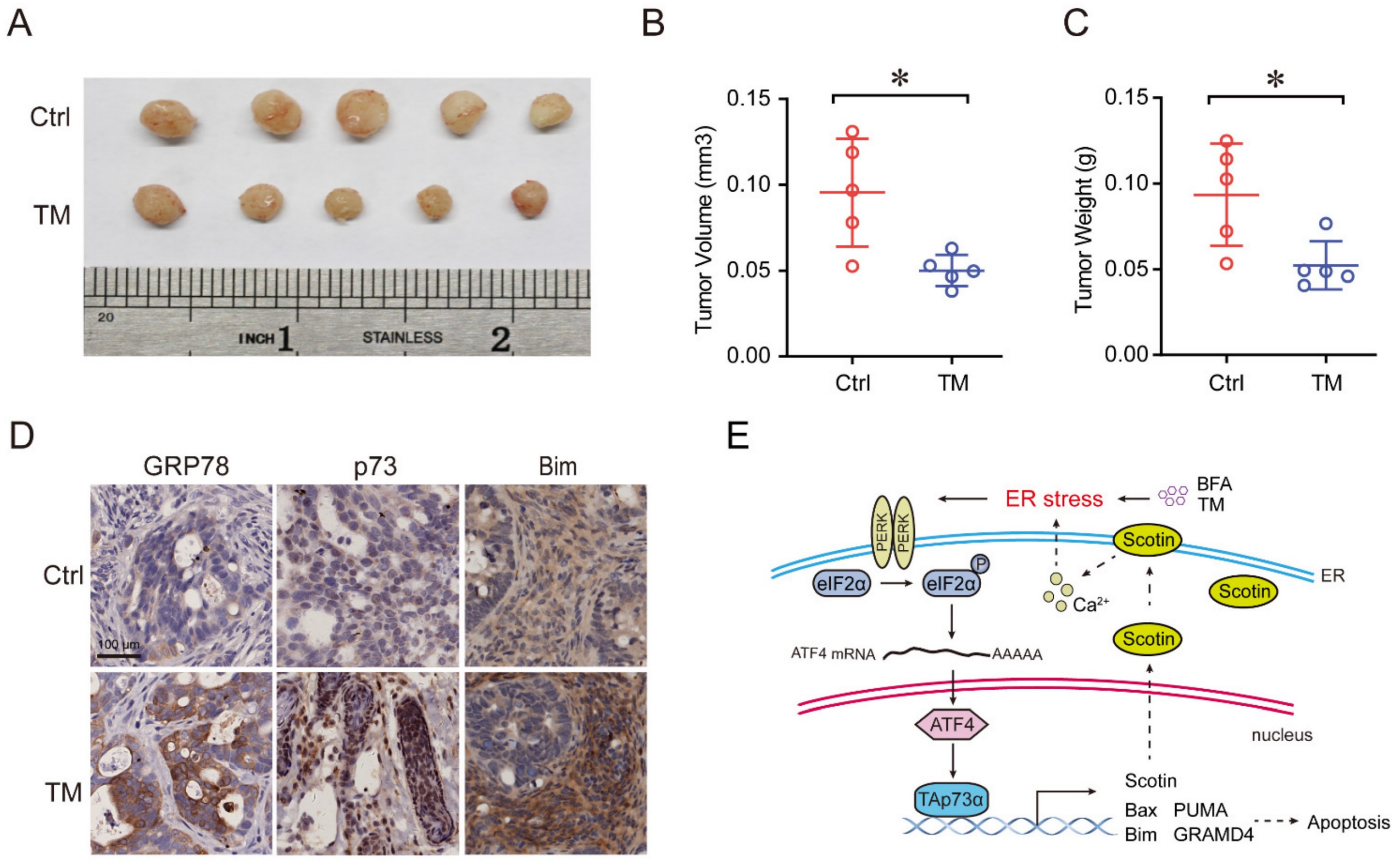
** Activation of ER stress inhibits colorectal cancer growth and promotes apoptosis *in vivo*.** (A) Colon cancer PDX model mice were treated with or without TM (0.25 mg/kg, 3 times a week) for three weeks. Tumors were harvested for imaging, and measurement of volume (B) and weight (C) and were then embedded in paraffin for IHC analysis (D). (E) A working model showing that ER stress activates TAp73α via PERK-ATF4 signalling to promote apoptosis.

To further verify the effect of ER stress on tumor growth *in vivo*, we established a colon cancer patient-derived xenograft model (PDX), in which a frameshift mutation was found at the R282 site in p53 protein. As shown in Fig. 5A-5C, treatment with the ER stress inducer, TM, significantly reduced the tumor volume and weight. Furthermore, IHC assays showed that the expression of the p73 protein was upregulated, concomitant with that of its target Bim and the ER stress marker GRP78 after TM treatment (Fig. 5D). Together, these results indicate that activation of ER stress inhibits colon cancer growth and induces apoptosis *in vivo*.

## Discussion

The endoplasmic reticulum (ER) is an important organelle in eukaryotic cells that connects the membrane, nucleus, and cytoplasm, and is the site of many important functions, especially the synthesis, folding and modification of secreted and transmembrane proteins 17, 18. Numerous external factors and intracellular events, as well as nutrient fluctuations, can disrupt the protein-folding capacity of the ER, causing the accumulation of unfolded or misfolded proteins, a condition referred to as ER stress, which activates the ER stress response (e.g., UPR) to decrease the biosynthetic rate of secretory pathway components by downregulating the expression of genes encoding secreted proteins. ER stress and UPR activation have important roles in tumor promotion and tumor suppression 11. On the one hand, ER stress promotes tumor cell survival, proliferation, and migration, which frequently correlate with advanced-stage cancers and chemoresistance 19, 20; on the other hand, prolonged ER stress mediates cancer cell death. Notably, the tumor suppressor p53 can also be activated and promote apoptosis in response to stress. A previous study indicated that p53 expression is significantly increased in response to ER stress and participates in ER stress-induced apoptosis 21. However, we found that ER stress can also induced apoptosis in p53 null colon cancer cells, suggesting that a p53-independent mechanism of ER stress-induced apoptosis might exist. In this study, we identified TAp73α, a homologue of p53, playing an essential role in ER stress-induced apoptosis. Knockdown of TAp73α restored ER stress-induced apoptosis in p53 null colon cancer cells.

TAp73α, is considered a tumor suppressor gene due to its high structural similarity with p53. TAp73α functions in tumor suppression mainly through apoptosis. Apoptosis is mediated by the accumulation of the cytoplasmic protein Bax on the outer mitochondrial membrane 22. TAp73α directly transactivates Bax expression to induce apoptosis. In addition, TAp73α transactivates key proapoptotic genes (Bim, Noxa, and PUMA), which induces mitochondrial accumulation of Bax and the release of cytochrome C 23, 24. TAp73α also transcriptionally activates GRAMD4, which translocates from the nucleus to mitochondria and then induces apoptosis 25. It has been reported that TAp73α promotes cell death by inducing the expression of death receptor CD95 7. Notably, TAp73α can directly transactivate Scotin, a target of the p53 protein. The Scotin protein level increases with a concomitant increase in the intracellular calcium level, which activates ER stress and leads to apoptosis 8. In this study, we found that ER stress can in turn activate TAp73α expression, suggesting a possible positive feedback loop between ER stress and TAp73α (Fig. 5E).

It has been documented that TAp73α expression and activity were regulated by numerous signalling pathways 23. Herein, we identified that PERK, not ATF6α or IRE1α, activates TAp73α mRNA and protein expression. PERK phosphorylates eIF2α at serine 51 to increase the translation of ATF4, which is a stress-induced transcription factor that controls the expression of many adaptive genes that allow cells to overcome stress. Our previous study demonstrated that ATF4 responds to cell migration induced by the oncogene HER2 via upregulating ZEB1 26. On the contrary, ATF4 also promotes apoptosis under persistent stress conditions 27. Glutamine depletion-induced ER stress promotes apoptosis by ATF4 dependent, but p53-independent, induction of PUMA, Noxa, and TRB3 expression in MYC-transformed cells 28. Previous reports demonstrated that ciclopirox activates PERK-ATF4 signalling to drive cell death in colon cancer 29. Consistent with this finding, we found that ATF4 is involved in PERK-mediated TAp73α expression and apoptosis in p53 null cells. We further showed that ATF4 can directly activate the transcription of TAp73α, consistent with previous research 30. Further evidence proved that ATF4 is responsible for ER stress-mediated TAp73α upregulation and apoptosis. In addition, the ER stress-activated transcription factor XBP1s can inhibit TAp73α expression to promote colon cancer cell proliferation 31, indicating that TAp73α expression is regulated by different branches of ER stress or UPR signalling.

Considerable clinical research evidence indicates that inactivation of tumor suppressor genes (TP53, BRCA1, and PTEN) or activation of oncogenes (Myc, Ras, and EGFR) leads to a robust increase in the metabolic demands of cancer cells 32, resulting in a rapid increase in protein synthesis and then inducing ER stress or activation of an adaptive UPR pathway, which promotes cancer progression. In contrast, activation of the apoptotic response to ER stress is often used in cancer therapy. In this study, we demonstrated that ER stress upregulated p73 and its target Bim in a p53 mutant PDX model. Together, our findings identified the PERK-ATF4 axis as a novel upstream regulator of TAp73 in ER stress-induced cancer cell apoptosis. Therefore, the induction of prolonged ER stress by novel compounds and therapeutic strategies is a useful strategy for tumor therapy.
